# Memory and Executive Screening (MES): a brief cognitive test for detecting mild cognitive impairment

**DOI:** 10.1186/1471-2377-12-119

**Published:** 2012-10-11

**Authors:** Qi-hao Guo, Bin Zhou, Qian-hua Zhao, Bei Wang, Zhen Hong

**Affiliations:** 1 Department of Neurology and Institute of Neurology, Huashan Hospital, State Key Laboratory of Medical Neurobiology, Shanghai Medical College, Fudan University, Shanghai 200040, China; 2 Translational Research Informatics Center, Foundation for Biomedical Research and Innovation, Kobe, Japan

**Keywords:** Mild cognitive impairment (MCI), Amnestic MCI-single domain (aMCI-sd), Amnestic MCI-multiple domain (aMCI-md), Alzheimer’s disease (AD), Memory and Executive Screening (MES), Mini-Mental State Examination (MMSE)

## Abstract

**Background:**

Mild cognitive impairment (MCI), defined as a transitional zone between normal cognition and dementia, requires a battery of formal neuropsychological tests administered by a trained rater for its diagnosis. The objective of this study was to develop a screening tool for MCI.

**Methods:**

One hundred ninety seven cognitively normal controls (NC), one hundred sixteen patients with amnestic MCI –single domain (aMCI-sd), one hundred ninety five patients with amnestic MCI-multiple domain (aMCI-md), and two hundred twenty eight patients with mild Alzheimer’s disease (AD) were evaluated by comprehensive neuropsychological tests and by the Memory and Executive Screening (MES).

**Results:**

Correlation analysis showed that the three indicators of the MES were significantly negatively related with age (P<0.05), yet not related with education (P>0.05). There was no ceiling or floor effect. Test completion averaged seven minutes (421.14±168.31 seconds). The receiver operating characteristics (ROC) analyses performed on the aMCI-sd group yielded 0.89 for the area under the curve (AUC) (95% CI, 0.85–0.92) for the MES-total score, with sensitivity of 0.795 and specificity of 0.828. There was 81% correct classification rate when the cut-off was set at less than 75. Meanwhile, the aMCI-md group yielded 0.95 for the AUC (95% CI, 0.93–0.97) for the MES-total score, with sensitivity of 0.87 and specificity of 0.91, and 90% correct classification rate when the cut-off was set at less than 72.

**Conclusion:**

The MES, minimally time-consuming, may be a valid and easily administered cognitive screening tool with high sensitivity and specificity for aMCI, with single or multiple domain impairment.

## Background

In China, the elderly above sixty years of age account for 13.26% of the population, and aging and Alzheimer’s disease (AD) have been significant public health problems [1]. Mild cognitive impairment (MCI), defined as a transitional zone between normal cognition and dementia [2], may be a target population for dementia early identification and intervention. MCI may occur in 15% of elderly patients [3]. Because of these large numbers and the absence of specific physiological markers, easier and earlier detection of cognitive impairment in the elderly population at minimal cost of time, resources, and expenses is increasingly important.

There are a variety of shorter or longer screening methods for MCI and mild dementia currently, such as the Seven minute neurocognitive screening battery(7MS) [4], the Memory Impairment Screen (MIS) [5], the clock drawing test [6], the cube-copying test [7], the DemTect [8], the AB Cognitive Screen (ABCS) [9], the AD8 [10], the Montreal Cognitive Assessment (MoCA) [11], the Short Cognitive Performance Test [12], the Addenbrooke’s Cognitive Examination Revised(ACE-R) [13], the Memory Alteration Test (M@T) [14], and the Memory Orientation Screening Test(MOST) [15]. There are differences among these tests for cognitive domain and item coverage, completion rate, administration times, diagnostic accuracy, ranging of total score and cut-off values. At present, no one screening test used for MCI is acknowledged internationally in the way that the Mini-Mental State Examination (MMSE) is for dementia [16].

Time length and complexity are the major concerns of brief tests. We found that the tests currently used, such as MoCA and ACE-R, take more than 10 minutes. Although some other non-comprehensive tests, e.g., the Clock drawing test and MIS, take a short time to administer, the simple scoring and administration methods without the necessity for training, their sensitivity and specificity in detecting MCI were relatively low [17]. AD, Frontotemporal lobar degeneration (FTLD) and vascular dementia (VaD) are the major types of dementia syndromes [18,19]. Memory impairment is often the earliest symptom of AD and executive function impairments are often the earliest symptoms of FTLD and VaD [20,21]. Therefore, more than orientation, attention, language and visuospatial function, tests focusing on memory and executive function could be the most sensitive for detecting early stages of MCI. As most tests are education-related, their application in populations with little education remains controversial. Tests independent of pencil and paper and without requirements for reading and writing could decrease this cultural bias.

Our goal was to develop a test that could be applied to detect MCI. Ideally, it could be administered in less than 10 minutes by a variety of clinical staff, be reliably and easily scored without the need of a complex algorithm or computer program, and be readily accepted by patients to detect the early stage of MCI. The test would be especially practical for those patients who require further evaluation or prompt initiation of medication and supportive services. No specialized testing materials would be required and it could be easily adapted for non-Chinese-speaking elderly. In this study, a brief test named Memory and Executive Screening was developed in which the instruction and content were acceptable for illiterate and low-educated people. In addition, this test didn’t require the participants to write. Therefore, we can expect that this is a test relatively independent of education.

## Methods

### Participants

A total of 796 (n=796) participants were recruited, including 197 (n=197) cognitively normal controls (NC), 116 patients (n=116) with amnestic MCI-single domain (aMCI-sd), 195 patients (n=195) with amnestic MCI-multiple domain (aMCI-md), and 228 patients (n=228) with mild Alzheimer’s disease (AD).

We recruited the NCs using cluster sampling in Jingansi Community, Shanghai, China from Jan 2009 to Jun 2009. The inclusion criteria for NC group were: age between 50 and 90; cognitively normal, based on the absence of significant impairment in cognitive functions or activities of daily living (ADL), no memory complaints or memory difficulties (verified by an informant); Clinical Dementia Rating (CDR) = 0 [22]; score of Hamilton depression rating scale [23] less than or equal to 12 on the 17-item scale in the preceding 2 weeks; and adequate visual and auditory acuity to allow cognitive testing. Participants with any significant neurologic disease and psychiatric disorders/psychotic features were excluded.

All patients were recruited from the Memory Clinic, Huashan Hospital, from Jun 2009 to Oct 2011.They underwent laboratory screening and cranial CT/MRI scan, with no clinically significant abnormalities in vitamin B12, folic acid, thyroid function (free triiodothyronine-FT3, free tetraiodothyronine-FT4, thyroid stimulating hormone-TSH, rapid plasma reagin-RPR, or treponema pallidum particle agglutination -TPPA.

Three hundred and eleven (n=311) participants were diagnosed as amnestic MCI according to the Peterson criteria [24]: (1) memory complaints and memory difficulties which are verified by an informant; (2) symptoms lasting more than 3 months; (3) total score of the Mini-mental state examination-Chinese version (CMMSE) [25] ≥ the cut-off score adjusted for education; objective memory impairment documented by scoring below the age- and education-adjusted cutoff on tests of episodic memory, including the Auditory Verbal Learning Test [26]; preserved basic ADL/minimal impairment in complex instrumental functions; (4) etiology unknown; (5) normal sense of hearing and sight; (6) has not met diagnostic criteria of dementia based on those of the National Institute of Neurological and Communicative Disorders and Stroke and the Alzheimer’s Disease and Related Disorders Association (NINCDS-ADRDA) [27]. Patients with aMCI were then differentiated as aMCI-sd and aMCI-md according to the impaired cognitive domains [28].

The mild AD patients (n=228) met the following criteria: Patients were diagnosed as probable AD according to the NINCDS-ADRDA; CDR=1; onset age ≥ 50 yrs; no obvious medical, neurological or psychiatric diseases or psychological dysfunction including anxiety and depression within the previous one month; no visual or auditory deficit.

Neurologists were in charge of the classification of normal, MCI and AD, taking the medical history, neuropsychological assessment and neuroimaging results into consideration.

The study was approved by the Huashan Hospital Foundation Ethical Committee, and each subject signed an informed consent.

For the Memory and Executive Screening (MES), see Appendix 1. There are three indicators for cognition evaluation. One sentence with ten main points is remembered three times and free delay recalled two times. The summation of the five recall scores is MES-5R. This reflects instant and delayed memory and learning ability. The four subtests of the MES-EX are the category fluency test, the sequential movement tasks, conflicting instructions task and Go/No-go task. This reflects executive function. The total possible score is one hundred, with fifty each for the MES-5R and MES-EX.

### Measures

Participants were given neuropsychological tests by a trained rater who was blind to diagnosis. Except for the MMSE and MES, a comprehensive neuropsychological battery that included memory, language, attention, executive function and visuospatial ability was used. The tests were as follows: the Auditory Verbal Learning Test (AVLT) [29], the Rey-Osterrieth Complex Figure Test (CFT) [30], the Boston Naming Test (BNT; the 30-item version) [31], the Animal Fluency Test (AFT) [32], the Symbol Digit Modalities Test(SDMT) [33], the Trail Making Test-A and B (TMT-A, TMT-B) [34], the Stroop Color-Word Test (SCWT) [33], the Similarity test [35], the Clock-drawing test [6], the Clinical Dementia Rating (CDR) [22] and Center for Epidemiologic Studies Depression scale (CESD) [36]. All tests have been proven to have a good reliability and validity with those of a Chinese cultural background. Each patient with MCI or AD received a CT/MRI examination.

### Statistical analysis

Chi-square analysis was adopted for ordinal data. Overall differences among the four groups (aMCI-sd, aMCI-md, mild AD and NC groups) were assessed with one-way analysis of variance (ANOVA). Post hoc pairwise comparisons between groups were assessed using the LSD test. The level of significance was set at α = .05. Pearson correlation was used to evaluate the relationship analysis. Receiver operating characteristic curves were used to assess the sensitivity, specificity and cut-off score. Associations of the MMSE and MES with the dichotomous clinical diagnoses were examined by using ROCs. The area under the ROC curves (AUC) was used as an overall index of performance of the screening tests. The AUCs and their standard errors were calculated using the method of Hanley and McNeil [37].

## Results

### Baseline characteristics

The comparison of general information and standard neuropsychological tests among the four groups is presented in Table 1. There were no significant differences in age, sex and education among the four groups (P>0.05).No significant differences were seen for MMSE between the aMCI-sd and aMCI-md groups, for delayed memory between the aMCI-md and mild AD, or for BNT, CFT-Copy, CWT-CR and TMT-part B between the aMCI-sd and NC groups (p>0.05). The testing confirmed the clinical features of patients with aMCI-sd and aMCI-md.

**Table 1:** Demographics and Standardized Neuropsychological Tests for the 4 Groups [ mean (standard deviation)]

**Index**	**NC (n=197)**	**aMCI-sd (n=116)**	**aMCI-md (n=195)**	**AD (n=228)**	**F(P)**
Age	68.84 (7.70)	70.04 (9.13)	70.27 (8.75)	70.19 (9.17)	1.164 (0.323)
Education	9.42 (5.17)	9.66 (4.85)	9.14 (5.19)	9.21 (4.98)	0.313 (0.816)
Sex^1^	110:89	53:63	96:100	129:102	1.813 (0.143)
MMSE	27.05 **(**2.11**)**	25.59 **(**2.61**)** **	25.17 **(**2.72) $$	19.09 (2.58)##	414.451 (<0.001)
AVLT-delayed recall(M=12)	5.30 (2.01)	1.19 (1.31) ††**	1.01 (1.27)	0.27 (0.83)##	176.26 7(<0.001)
BNT(M=30)	23.56 (4.55)	23.56(4.75) ††	21.08 (3.51) $$	18.01 (5.47)##	21.787 (<0.001)
SDMT	41.40 (11.98)	37.40 (10.57) ††	29.37 (13.48) $$	20.16 (11.89)##	33.441 (<0.001)
CFT-Copy(M=36)	33.22 (3.42)	32.86 (3.13) †	29.73 (7.54) $	24.74 (9.31)##	22.917(<0.001)
CFT-delayed recall(M=36)	15.47 (5.55)	11.69 (7.39) †	9.07 (5.58) $$	4.15 (3.34) ##	38.909(<0.001)
CWT-CR(M=50)	45.73 (3.94)	42.96 (9.61) †	38.70 (10.43) $$	29.50 (13.68)##	33.370(<0.001)
TMT-partA(s)	55.08 (20.78)	64.43 (25.25) ††	84.66 (36.57) $$	118.30 (48.31) ##	36.214(<0.001)
TMT-part B(s)	155.89 (63.99)	172.08 (68.55)††	250.37(107.90)$$	331.28(131.65)##	32.921(<0.001)
CESD	11.43(2.24)	13.67(3.12)	10.30(2.79)	10.64(3.06)	1.329(0.266)

Comparison between NC group and aMCI-sd group is marked behind ‘aMCI-sd group’; ** P<0.01.

Comparison between aMCI-sd group and aMCI-md group is marked behind ‘aMCI-sd group’; † P<0.05; †† P<0.01.

Comparison between aMCI-md group and AD group is marked behind ‘aMCI-md group’; $ P<0.05; $$ P<0.01.

Comparison between NC group and AD group is marked behind ‘AD group’. # P<0.05; ## P<0.01.

AVLT: Auditory Verbal Learning Test; BNT: Boston Naming Test; SDMT: Symbol Digit Modalities Test; CFT: Rey-Osterrieth Complex Figure Test; CWT-CR: Card C right of Stroop Color-Word Test; TMT: Trail Making Test; CESD: Center for Epidemiologic Studies Depression.

^**1**^ chi-square test, M=maximum.

### Essential features of MES

#### Demography factors and MES

Correlation analyses were carried out for the NCs. Age was significantly related with the three indicators of MES (p<0.05). When a person was older, s/he obtained a lower score. According to age, four subgroups were determined for NCs. There were 23 persons aged 50–59, 87 aged 60–69, 74 aged 70 –79, and 13 aged 80–89. The total scores for the MES were 84.0, 83.0, 80.5, and 77.9, respectively. There were distinct differences among subgroups (F=2.972, P=0.033), yet education level had no relationship with the test (p>0.05). No differences were found between male and female in the total scores and factor scores of the MES. In contrast, age and education significantly correlated with the MMSE score (correlation coefficients were −0.233 and 0.304, respectively, p<0.01).

#### The relationship of MES-5R, MES-EX and MES

Correlation analyses were done for all participants. The correlation coefficients were 0.892 for MES-5R and MES total score (p<0.01), 0.882 for MES-EX and MES total score (p<0.01), and 0.573 for MES-5R and MES-EX (p<0.01).

#### The relationship of MES and standard psychological tests

The coefficients were 0.663 for MES-5R and AVLT-total score (p<0.01), 0.523 for MES-5R and CFT-delay recall, 0.554 for MES-EX and the SCWT- interference effects, and 0.381for MES-EX and time scores of the TMT-part B (p<0.01).

#### Ceiling and floor effects

For NCs, the proportions obtaining the maximum score on the MES-5R, MES-EX and MES total were 4.1%, 20.3% and 2.5%. For mild AD subjects, 2.2% scored zero in the subtest of MES-5R, but no one scored zero in the MES-EX and MES-total. The scores of MCI patients were intermediate between the NC and AD groups. This demonstrated that there were no obvious ceiling and floor effects.

#### Test administration times

The average administration times were 421.14±168.31 seconds, about seven minutes, for the MES test, and 363.20±144.47 seconds, about six minutes, for the MMSE.

#### Completion rate

For the elderly from the community who were the NCs, 4% rejected finishing the cognitive testing, but when subjects were willing to finish the MMSE, they also finished the MES. The outpatients were examined by a trained rater in the neuropsychological department. The completion rate of the patients with MCI and mild AD was 100%.

#### Reliability

The data were collected twice from a subsample of 30 participants (patients and controls) tested, 29.1(5.8) days apart on average. The mean change in MES total scores from the first to the second evaluation was 4.7(5.8) points, and the correlation among the scores of the 5 briefly trained raters evaluations was high (Pearson r = 0.92, P<0.001).

### Comparisons of MES scores among four groups

The scores of the four groups are presented in Table 2. The results of patients with aMCI were intermediate between the NC and AD groups. For the aMCI-sd group, the memory functions declined obviously, while the decrease of executive function was relatively slight. For the aMCI-md group, the executive function was inferior to that of the aMCI-sd group, as was memory function. In general, the cognitive deficits of the aMCI-md group were more serious than those of the aMCI-sd group. The pattern of cognitive deficits for aMCI-md was similar to that of mild AD.

**Table 2:** Comparisons of MES score among the four groups

**Index**	**NC** (**n**=**197**)	**aMCI**-**sd** (**n=116)**	**aMCI-md (n=195)**	**AD (n=228)**	**F(P)**
MES-5R	36.64 (7.66)	24.67 (6.72) ††**	21.28 (8.62) $$	13.59 (6.71)##	339.125 (<0.001)
MES-EX	45.60 (3.89)	41.81 (5.07) ††**	35.05 (6.99) $$	24.36 (9.98)##	340.003(<0.001)
MES total	82.25 (9.40)	66.49 (8.16) ††**	56.33 (11.30) $$	37.96 (12.20)##	628.306(<0.001)

Comparison between NC group and aMCI-sd group is marked behind ‘aMCI-sd group’; ** P<0.01.

Comparison between aMCI-sd group and aMCI-md group is marked behind ‘aMCI-sd group’; †† P<0.01.

Comparison between aMCI-md group and AD group is marked behind ‘aMCI-md group’; $$ P<0.01.

Comparison between NC group and AD group is marked behind ‘AD group’. ## P<0.01.

### ROC analyses of MES and MMSE

As shown in Table 3, according to the area of the ROCs, the MES total score was more helpful for aMCI-sd and aMCI-md discrimination than was the MMSE. The MES-5R identified aMCI-sd better than the MES-EX, whereas for aMCI-md, the MES-EX was superior to the MES-5R.

**Table 3:** ROC analysis of MMSE and MES

	**Index**	**ROC area under the curve**	**95% Confidence Interval**	**Cut-off**	**Sensitivity (%)**	**Specificity (%)**	**Correct classification rate(%)**
NC vs aMCI-sd	MMSET	.669	.606–.732	≤27	67.7	61.2	65.2
	MEST	.893	.858–.928	≤75	79.5	82.8	80.8
NC vs aMCI-md	MMSET	.715	.663–.767	≤27	67.7	70.0	68.9
	MEST	.956	.938–.974	≤72	87.8	91.3	89.5
NC vs AD	MMSET	.985	.976–.994	≤24	91.8	98.2	95.3
	MEST	.998	.996–1.000	≤62	99.0	97.8	98.4

In terms of the MES total score, 75–62 appears to be the range for patients with aMCI. Subjects exceeding 75 were usually considered as NCs, and subjects scoring less than 62 may be suspected as having dementia. In the range 75–62, the lower the score, the more likely the diagnosis of aMCI-md, while the higher the score, the more likely the diagnosis of aMCI-sd.

**Figure 1:**
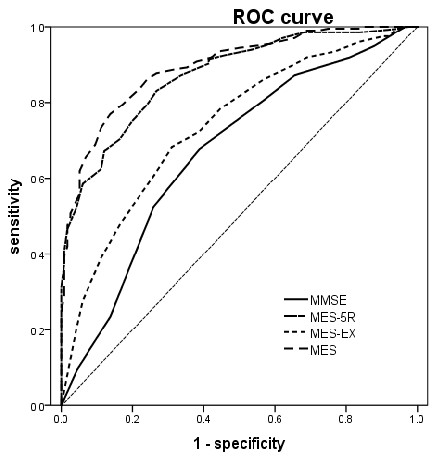
ROC curve of NC vs aMCIsd.

The ROC analyses performed on the aMCI-sd group yielded 0.89 for the area under the curve (AUC) (95% CI, 0.85–0.92) for the MES-total score, with sensitivity of 0.795 and specificity of 0.828 , and 81% correct classification rate when the cut-off was less than 75. The MMSE had 0.66 AUC (95% CI, 0.60–0.73), with sensitivity of 0.67 and specificity of 0.61, and 65% correct classification rate when the cut-off was less than 28. The AUC of the MES-total score was significantly higher than that of the MMSE (Z=6.948,P < 0.0001). The ROC graphs are presented in Figure 1.

**Figure 2:**
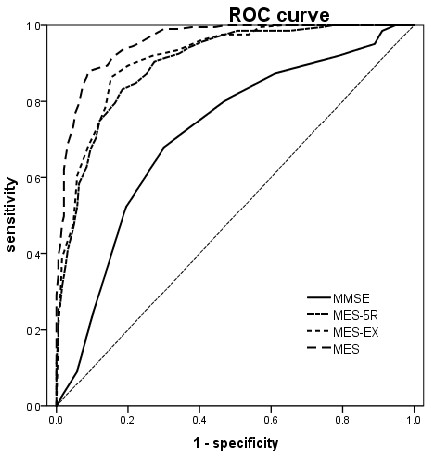
ROC curve of NC vs aMCI-md.

The ROC analyses performed on the aMCI-md group yielded 0.95 for the area under the curve (AUC) (95% CI, 0.93–0.97) for the MES-total score, with sensitivity of 0.87 and specificity of 0.91, and 90% correct classification rate when the cut-off was less than 72. The MMSE had 0.71 AUC (95% CI, 0.66–0.76), with sensitivity of 0.67 and specificity of 0.70, and 69% correct classification rate when the cut-off was less than 28. The AUC of the MES-total score is significantly higher than that of the MMSE (Z=9.732,P < 0.0001). The ROC graphs are presented in Figure 2.

**Figure 3:**
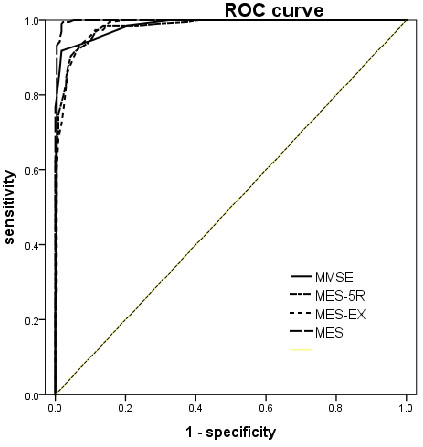
ROC curve of NC vs Mild AD.

The ROC analyses performed on the mild AD group yielded 0.99 for the area under the curve (AUC) (95% CI, 0. 99–1.00) for the MES-total score, with sensitivity of 0.99 and specificity of 0.97, and 98% correct classification rate when the cut-off was less than 62. The MMSE had 0.985 AUC (95% CI, 0.97–0.99), with sensitivity of 0.91 and specificity of 0.98, and 95% correct classification rate when the cut-off was less than 25. The AUC of the MES-total score is significantly higher than that of the MMSE (Z=2.866,P =0.0042). The ROC graphs are presented in Figure 3.

## Discussion

The results showed that the MES may be a suitable screening method for MCI and mild dementia. First, time was saved as the most involved cognitive domains were evaluated selectively; second, the score range for memory and executive function was large enough to identify MCI; third, it was independent of pencil and paper, and reading and writing skills were not required of subjects. Hence, education was not a factor with the MES score.

In order to reach our aim, much preliminary work was carried out. With regard to the memory materials in the neuropsychological tests, the sentence, rather than word list, is more suitable for the illiterate and low-educated people. Lengths of sentences and numbers of trials and recalls were determined by careful consideration and repeatable pilot studies. Second, as implicit memory is relatively preserved for mild AD patients [38], memory materials chosen were unfamiliar. Person (Li Xiao-ming) and place names (He-xi town and Yong-an county) were imaginary. Third, auditory memory material appeared to be more sensitive than visual material for the Chinese elderly [26]. Delayed recall of episodic memory may be the most sensitive predictor for AD, but only long delay recall, easily producing floor effects, was not applied to evaluate severity of memory damage and cognitive change at follow-up. Accordingly, in our study, the summation of immediate and delayed recall performances was the indicator for the memory factor.

For patients with AD, executive function was another cognitive domain involved in addition to memory function, and the initial damaged domain for FTLD and VaD patients. In the beginning of the process of compiling the MES, we searched for various executive memory tests independent of pencil and paper in the literature [35,39-41]. The initial tests included the Category Fluency Test,the Object Figure Naming and Sorting Test, the Conflicting Instructions Task, the Go/No Go Task, the Sequential Movement Tasks, the Oral Symbol Digit Modality Test, Stroop Color Words Test, the Similarities Test, the Proverbs Test, the Wisconsin Card Sorting Test, the California Cards Sorting Test, the Tower of London or Tower of Hanoi, and the Paced Auditory Serial Addition Test. Preliminary application and verification were undertaken. Considering the rate, reliability and validity of accomplishment, we selected the Conflicting Instructions Task, the Go/No Go Task, the Sequential Movement Tasks and the Verbal Fluency Test as the subtests of the MES-EX. The tests could be used for the two usual components of executive function including the set shift and dominant inhibition. The MES ratio (MES-5R/MES-EX) may apply for distinguishing between AD and FTLD or VaD. There is one point that we should emphasize, namely, that the four subtests of the MES-EX are similar to the four tests of the frontal assessment battery( FAB) [41] in the meaning of the tests, but the concrete operations, procedure and scoring standard are different.

The MES was related to aging. Many studies have shown that executive function may decrease with increasing age [42,43]. Level of education was not related to MES performance. In western countries, the education level for the elderly is generally high, and the focus of researchers may be the effects of age and gender on neuropsychological tests. However, in a developing country like China, illiterate persons remain a significant proportion of the population. According to the sixth population censuses in 2010, of the total population, the proportion of persons with education exceeding university level accounted for 8.7%, senior high school for 13.7%, junior high school for 37.9%, and primary school for 26.2%, while the illiteracy rate was 13.5%. The numbers of elderly with low levels of education was therefore expected to be large. As the result, it was necessary to compile tests suitable for people with low education levels. In our sample, there were quite a lot of persons with low education levels, or even illiterate, and also individuals with high education levels and university careers. The statistical results showed that the MES was independent of education and knowledge. As the subtests were the items independent of reading and writing, the MES may be used for cross-cultural comparison of different countries.

At present, there have been few studies about the MCI subtypes of single-domain and. multiple-domain mild cognitive impairment [44]. Newer prospective studies show that multiple-domain MCI (particularly amnestic) confers greater risk of progression to dementia than single-domain MCI, even when examining multiple domains of MCI [45-47]. Those with single-domain MCI and naMCI (non-amnestic MCI) have a relatively high rate of reversion to normal cognition [48,49]. Mitchell et al. [50]discovered that of the multi-domain MCI group, 59% progressed to dementia and only 5% improved. By contrast, in pure aMCI, only 18% progressed and 41% improved by two-year follow-up. These findings may simply reflect a threshold/definitional effect, in that multiple-domain impairment represents more advanced disease than single-domain impairment and is closer to the dementia threshold, that is, the outcome of interest is very similar to the predictor. As a result, differentiation of MCI subtypes has been necessary. The MES test, as a tool to identify single-domain and multiple-domain subtypes at a given point, may be helpful for the prognosis of MCI.

Total time for MES administration and scoring averages approximately seven minutes, similar to the time for the MMSE or DemTect and notably less than that of the MoCA and the seven minute neurocognitive screening battery (which actually needs 12 minutes).

As a screening test, the content of items of the MES is different from other common neuropsychological tests such as the MMSE, the Mini-Cog, and the ADAS-cog [51,52], and could be administered together with those tests. As a part of the annual health check for the elderly, the MES could also be performed alongside other routine measurements (height, weight, and blood pressure) as a measurement of objective cognitive function. The MES would increase the probability of earlier diagnosis and improve ability to monitor change over time and treatment response in clinic outpatients. The feasibility of the MES as a follow-up tool has been validated in the process.

## Appendix 1: Memory and Executive Screening (MES)

Q1. The rater should read out the following sentence and have the subject repeat (Do not let the subject read the sentence). [Li] [Xiao-Ming] has [two][gray][puppies], and lives at [No. 58], [ He-xi][town], [Yong-an][county]. There are a total of 10 key points, they are the words in the square brackets. Subject gets 1 point for each key point he/she answers correctly. The subject does not score a point if his/her answer is only partially correct (e.g., Saying “Shao-Ming” instead of “Xiao-Ming”). The subject is allowed to repeat the key points in reverse orders (e.g., saying “Li Xiao-Ming’s two gray puppies are gray”). Repeat the sentence two more times and write down the subject’s answer.Note: 1).Requires rater to read the sentence continuously. Do not respond to any of the subject’s questions in between. 2). After the third time recalling, inform the subject “Please remember the sentence, I will ask you to repeat it later.” 3). The subject does not need to learn the third time if he/she answered all the key points correctly in the first or second time.Q2. ”Please generate all the things you can think of that can be used or seen in the kitchen”, count down 30 seconds and write down all the subject’s answers, even if there are more than 10.Q3. Conflicting Instructions: “When I tap twice, you tap once, and when I tap once, you tap twice.” In order for the subject to understand the rule, please demonstrate: The practitioner taps once, and the subject should tap twice; the practitioner taps twice, and the subject should tap once. Every tap should be about 2 seconds. Finish the series of number below: 1-1-2-1-2-2-2-1-1-2-1-2-2-1-1. Score method: Mark the ones he/she tapped wrong, minus 1 point for each mistake. Range score is 0–10.Note: 1). The numbers should be tapped continuously and equally. Finish the task in 30 seconds. 2). Make sure the subject understands the rules completely before starting. Once you begin, tap the numbers equally and do not respond to any interference until the task is done. 3). Avoid suggesting to the subject whether to tap or not. 4). The subject only scores when his/her finger touches the table. The tap does not count if his/her finger stops half way. 5). To avoid hurting the subject’s finger while tapping, subject can also choose pounding the table instead.Q4 Short delayed recall: 4^th^ time.Rater does not repeat the above sentence and asks subject to recall the previous sentence that was learned before.Q5. Have the subject use his/her hand to imitate the following action one hand at a time. Imitate every action once.Step 1: Subject uses dominant hand to imitate the action, and complete with single hand. Subject scores 2 points if correctly done.Step 2: If the action is not imitated correctly, the practitioner can repeat the action one more time. Subject scores 1 point if correctly done.Step 3: Subject uses non-dominant hand to imitate the action without the practitioner demonstrating with non-dominant hand. Subject scores 2 points if correctly done.Step 4: If the action is not imitated correctly with subject’s non-dominant hand, the practitioner can repeat the action one more time with non-dominant hand. Subject scores 1 point if correctly done.Note the similarity and sequence of the shape of gesture in every movement. 2 points for each action that is completely correct first imitation, and one point if a second repetition is needed (no third repetition).

1) Use thumb to touch other four fingertips in order

2) Put thumb between index finger and middle finger–scissor shape

3) Put wrist on same side eye(Telescope-like gesture)–same side ear(Listen-like gesture)–mouth(Drink-like gesture)

4) Do a cross (touch forehead, chest, contralateral shoulder and ipsilateral shoulder by order)

5) Luria action. Instructions: Make a fist, slice down with the edge of your palm, then close your fingers and put the back of your hand flat on the table.

Q6. Inhibitory Control Test (Go/No-go test): “When I tap once, you tap once, and when I tap twice, you don’t tap.” In order for the subject to understand the rule, please demonstrate: The practitioner taps once, and the subject should also tap once; the practitioner taps twice, and the subject should not tap. Start the test after the subject understands the rule. If there are errors in the process, do not remind the subject. Every tap should be about 2 seconds. Finish the series of number below: 1-2-1-2-1-1-2-2-1-1-2-1-2-1-2. [Score method] Mark the ones he/she tapped wrong, minus 1 point for each mistake. Range score is 0–10.Note: The numbers should be tapped continuously and equally. Do not respond to any of the subject’s questions in between. Finish the task in 30 seconds.Q7 Long delayed recall: (5^th^ time).Ask the subject to once again recall the sentence that was learned before.

## Conclusions

The MES may be a highly sensitive and specific cognitive screening tool that is valid, easy to administer, and minimally time-consuming. It can be applied as a screening tool for large epidemiological surveys. Because the score range and gradient change of test difficulty are large enough, it may be suitable to evaluate cognitive changes during therapy for outpatients. Further longitudinal studies will be undertaken to investigate some issues, such as the follow-up value, discrimination of aMCI and naMCI, the relationship of MES score and MRI features (hippocampus or cerebral atrophy), and cerebral spinal fluid biomarkers. Additionally, there are some cognitive domains that the MES could not measure. Whether some atypical ADs, such as those manifesting of language deficit or visual spatial impairment, will miss being diagnosed, is worth further investigation.

## Abbreviations

MES: Memory and executive screening; MMSE: Mini-mental status examination; CMMSE: Mini-mental status examination, Chinese version; MCI: Mild cognitive impairment; AD: Alzheimer’s disease; NC: Normal control; aMCI-sd: Amnestic mild cognitive impairment - single domain; aMCI-md: Amnestic mild cognitive impairment - multiple domain; naMCI: Non-amnestic mild cognitive impairment; AUC: Area under curve.

## Competing interests

The authors declare that they have no competing interests.

## Authors’ contributions

GQH participated in the design of the study, data collection and drafted the manuscript. ZB performed the statistical analyses and critically reviewed the manuscript. ZQH carried out the neurological evaluation and clinical diagnosis and critically reviewed the manuscript. WB critically reviewed the manuscript and helped to perform data collection. HZ participated in the design and coordination of the study and contributed to its final version. All authors read and approved the final manuscript.

## Pre-publication history

The pre-publication history for this paper can be accessed here:

http://www.biomedcentral.com/1471-2377/12/119/prepub
